# Peritoneal Mesonephric-Like Adenocarcinoma Arising in Endometriosis: Case Report and Review of the Literature Expanding the Spectrum of Extrauterine Mesonephric-Like Adenocarcinomas

**DOI:** 10.1177/10668969261437122

**Published:** 2026-04-16

**Authors:** Khaled Sabry Mohamed, Kareem Elfatairy, Jennifer Crow, Steven Holloway, Elena Lucas

**Affiliations:** 1 Department of Pathology, 12334 UT Southwestern Medical Center, Dallas, TX, USA; 2 Department of Radiology, UT Southwestern Medical Center, Dallas, TX, USA; 3 Department of Pathology, Texas Health Hospital Mansfield, Mansfield, TX, USA; 4 Department of Obstetrics and Gynecology, UT Southwestern Medical Center, Dallas, TX, USA

**Keywords:** mesonephric-like adenocarcinoma, mesonephric, endometriosis, peritoneal, extrauterine

## Abstract

**Background:**

Mesonephric-like adenocarcinoma (MLA) is a rare type of Müllerian carcinoma that poses significant diagnostic challenges, especially at extrauterine sites. Its morphologic and immunophenotypic overlap with other carcinomas can lead to diagnostic confusion or misclassification.

**Patient Presentation:**

We describe a 55-year-old woman with a history of endometriosis who developed peritoneal MLA infiltrating the bowel wall, accompanied by nodal and hepatic metastases. The tumor displayed diverse architectural patterns and was composed of cuboidal to columnar cells with moderate cytologic atypia and vesicular chromatin. Immunohistochemistry showed positivity for keratin, PAX8, and TTF-1, with focal positivity for GATA3 and luminal CD10, and focal weak ER staining in <5% of cells; PR was negative. Focal thyroglobulin expression was also present. Molecular testing revealed a *KRAS* p.G12V activating mutation.

**Discussion:**

MLA has a broad differential diagnosis and is often misinterpreted as other neoplasms. Accurate diagnosis requires an integrated assessment of morphology, immunophenotype, and molecular profile. The peritoneal location, particularly in association with endometriosis, raises the possibility of a peritoneal origin.

**Conclusion:**

Peritoneal MLA is exceptionally rare. Pathologists should consider MLA in the differential diagnosis of peritoneal tumors, particularly those arising in the setting of endometriosis, to ensure accurate classification and appropriate clinical management.

## Introduction

Mesonephric-like adenocarcinoma (MLA) was first described in 2016 and subsequently included in the 2020 WHO Classification of Female Genital Tumours. MLA displays morphologic and immunophenotypic similarities to mesonephric adenocarcinoma of the uterine cervix.^1,2^ However, accumulating evidence suggests a Müllerian origin with mesonephric transdifferentiation rather than a true mesonephric lineage.^1,3^

MLA is a rare tumor, accounting for approximately 1% to 3% of endometrial carcinomas^4–7^ and approximately 0.6% to 1.5% of ovarian epithelial tumors.^8,9^ Notably, the peritoneum as a potential site of origin is exceedingly rare and likely under-recognized. To date, only six peritoneal MLAs have been documented in the literature, including one case series and one institutional study.^10,11^ Herein, we describe peritoneal MLA arising in a patient with a history of endometriosis, with no adnexal or uterine masses identified on transvaginal ultrasound.

## Patient Presentation

**Figure 1.**
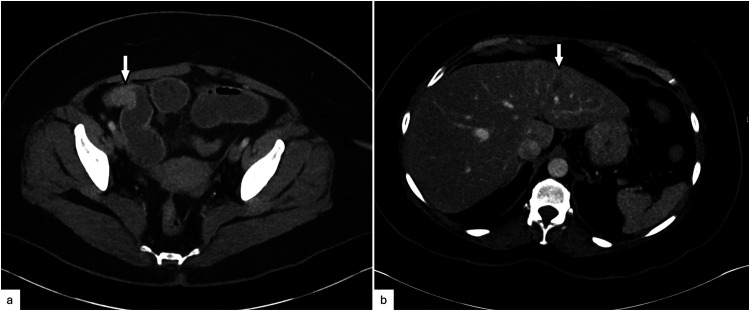
CT abdomen and pelvis composite images showing: (a) mild small-bowel dilation with a transition point at a stricturing focal circumferential wall thickening in an ileal loop (2.2 cm) (arrow); (b) a 1.1-cm hypoenhancing lesion (arrow) in the left hepatic lobe concerning for metastasis.

A 55-year-old woman presented with clinical features of bowel obstruction which were demonstrated on cross-sectional imaging along with multiple hepatic metastases (Figure 1A, B). She had a history of endometriosis, for which she underwent exploratory laparotomy more than 30 years earlier and a right salpingo-oophorectomy 15 years earlier. She presented at a community hospital with complaints of pain and rectal bleeding. She underwent a colonoscopy with terminal ileal biopsy. One week later, she was hospitalized due to a small bowel obstruction and underwent a right hemicolectomy and liver biopsy. Pathologic examination revealed stage IV adenocarcinoma involving the colon and small bowel with metastases to the liver and 7 of 22 lymph nodes.

The tumor cells were interpreted as positive for epithelial markers, keratin 7, PAX8, and TTF-1 (NKX2-1), focally positive for thyroglobulin, and negative for keratin 20, CDX2, podoplanin (D2-40), GATA3, and WT1. Molecular analysis demonstrated a microsatellite stable (MSS) tumor with a low tumor mutational burden (TMB: 6.3 mut/Mb) and multiple genetic alterations, including *KRAS* p.G12V (activating) and *ATM* p.S1092 mutations. No alterations were detected in *BRAF*, *MET*, or *RET* genes. Tumor tissue was also submitted for CancerTYPE ID molecular analysis (Biotheranostics), which revealed a 90% probability of renal cell carcinoma, though thyroid and endometrial origins could not be excluded (reported as 6% and <5% probability, respectively). The patient was started on carboplatin and paclitaxel chemotherapy for carcinoma of unknown primary.

The patient was referred to a cancer center for further evaluation where a diagnosis of carcinoma involving small bowel, appendix, lymph nodes, and adipose tissue, most compatible with metastatic thyroid primary, was rendered. However, extensive workup including various imaging studies failed to identify a definitive primary site. Neck ultrasound revealed a normal thyroid gland without nodules or lymphadenopathy. Transvaginal ultrasound showed no adnexal or uterine masses, although adenomyosis was noted.

The patient sought a second opinion at our institution. Review of the right hemicolectomy and liver biopsy slides revealed tumor transmurally infiltrating the colonic and small intestinal walls with involvement of the appendix. Seven lymph nodes contained metastatic deposits, and a concurrent liver biopsy was positive for metastatic carcinoma. Small foci of endometriosis, highlighted by ER and PR, were identified within the appendix and colonic peritoneum.

**Figure 2.**
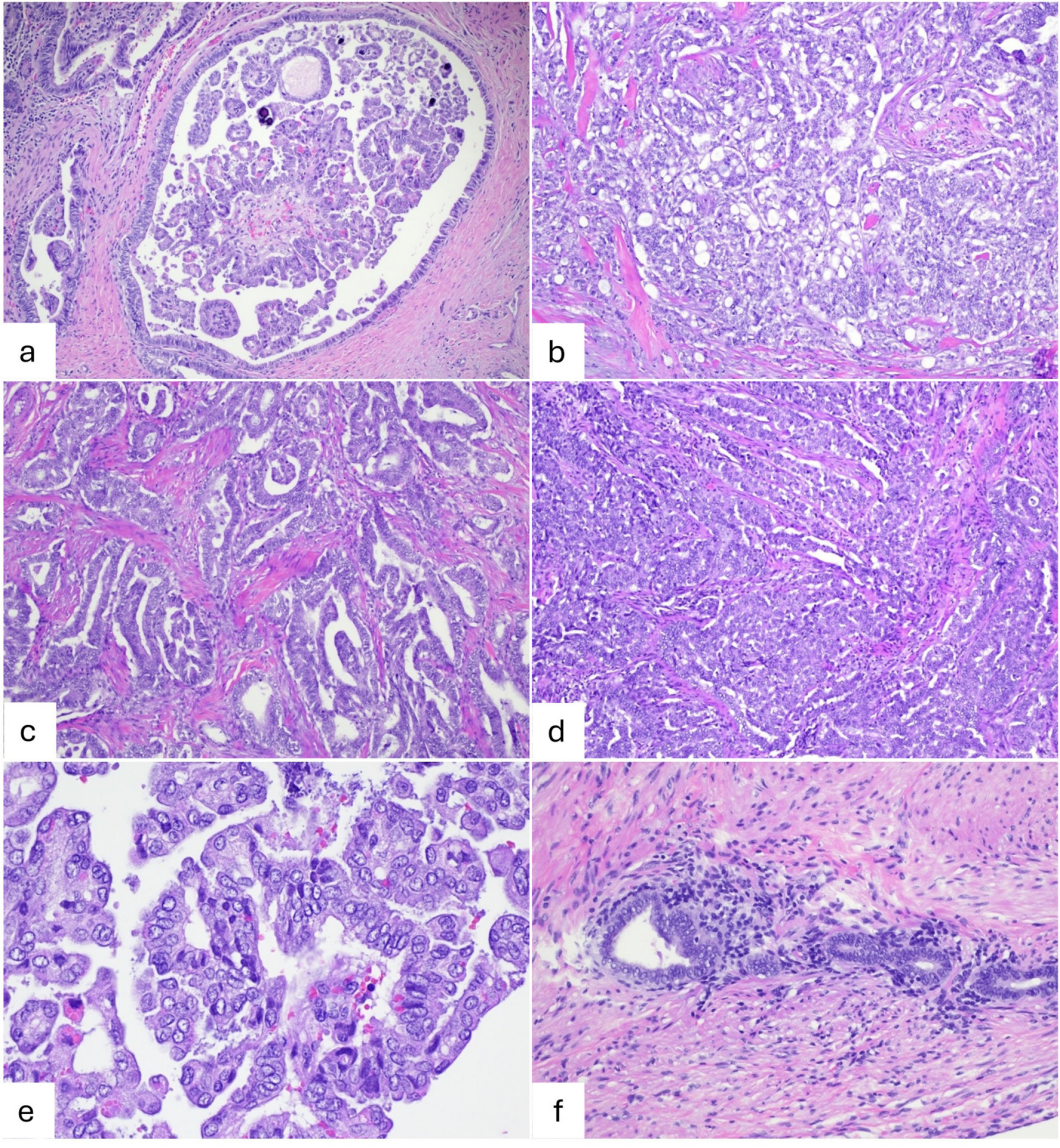
Histopathologic features of the tumor demonstrating multiple morphological patterns, including (a) papillary structures with psammomatous calcifications, (b) cribriform growth, (c) glandular, ductal, and glomeruloid formations, (d) retiform architecture, and (e) on high power view showing some of papillary thyroid carcinoma-like nuclear features such as overlapping, irregular nuclear membranes, and focal nuclear grooves and chromatin clearing. (f) An image of appendiceal wall shows endometriosis.

**Figure 3.**
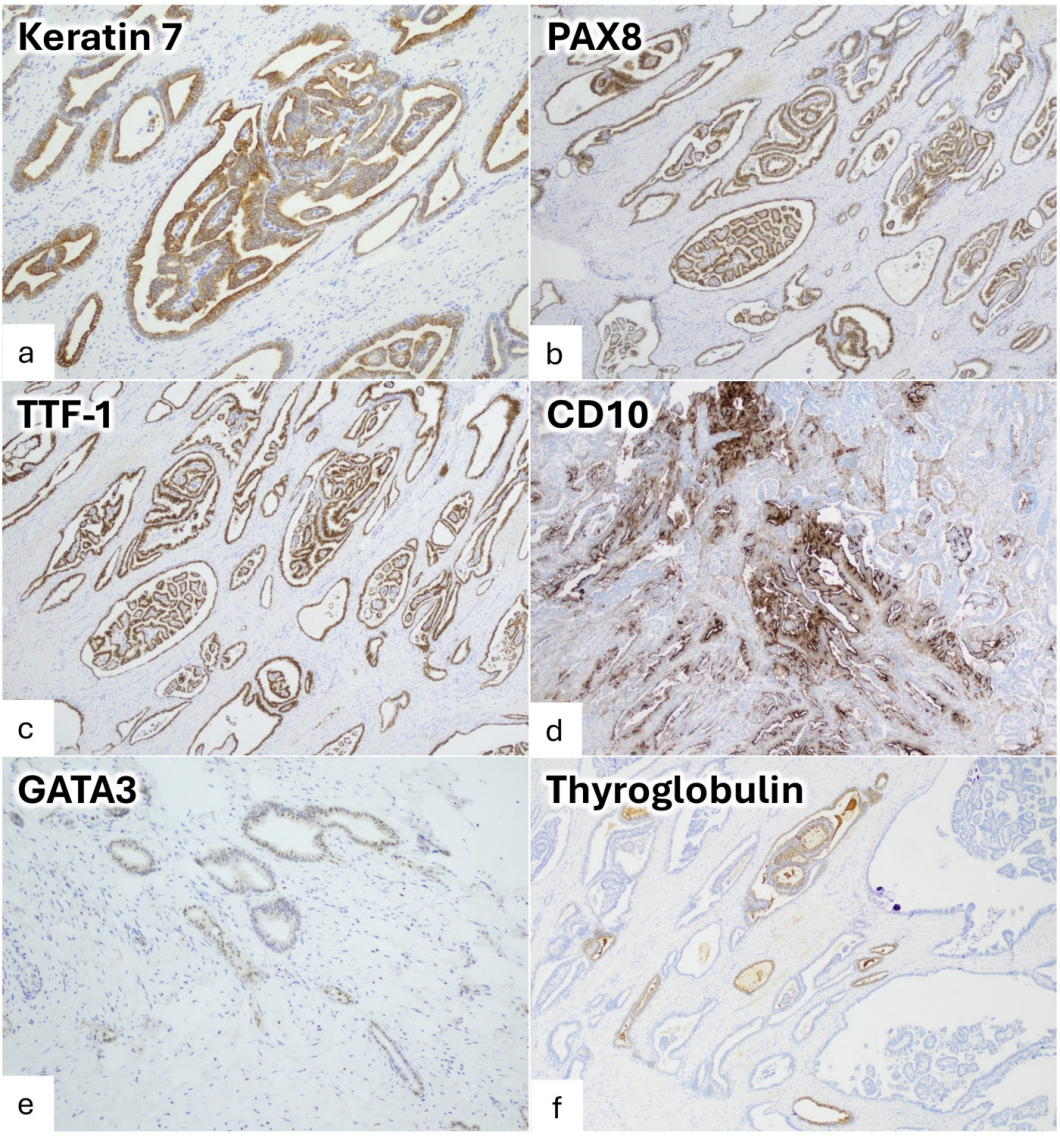
Selected immunohistochemical studies: (a) keratin 7 positive; (b) PAX8 positive nuclear staining; (c) TTF-1 positive nuclear staining; (d) CD10 showing focal luminal staining; (e) GATA3 shows focal positive staining; (f) thyroglobulin shows focal positivity.

The tumor cells were arranged in a variety of architectural patterns, including papillary, cribriform, glandular, ductal, glomeruloid, and retiform. Scattered psammomatous calcifications were present. The tumor cells were cuboidal to low columnar, with mild to moderate nuclear atypia, and vesicular chromatin (Figure 2A-F). Mitotic figures were increased. Immunohistochemically, the tumor cells were diffusely positive for keratin AE1/AE3, EPCAM, keratin 7, PAX8, and TTF-1; patchy positive for CD10 (luminal) and p16 (INK4a/CDKN2A); focally positive for GATA3 and very focally for thyroglobulin; and negative for keratin 20, CDX2, WT1, D2-40, and BRAF (Figure 3A-F). p53 demonstrated a wild-type expression pattern. ER and PR were essentially negative (focal weak ER staining was observed in <5% of cells).

Based on the combination of the morphological features, immunophenotype, molecular profile, and the presence of endometriosis, a final diagnosis of endometriosis-associated MLA was rendered.

## Discussion

MLA is a rare tumor type that was only recently described.^1,2^ Before its recognition, MLAs of the endometrium were misclassified as either endometrioid adenocarcinoma or, rarely, mesonephric adenocarcinoma arising in an unusual location, such as the uterine corpus.^2,3,12–14^ The diagnostic assumption of mesonephric adenocarcinoma was based on the tumor's presence in the deep myometrium, which was thought to arise from mesonephric remnants in the lateral uterine wall with extension to the endometrium.^13^ The tumors misclassified as endometrioid adenocarcinoma were noted to express TTF-1 and had a propensity for pulmonary metastasis, leading to the proposal that TTF-1 positivity was a worse prognosticator in low-grade endometrioid adenocarcinoma.^1,2,12,15^ Although MLA exhibits morphologic overlap with mesonephric adenocarcinoma and Müllerian carcinomas, especially endometrioid adenocarcinoma, it has become a distinct entity characterized by an increased risk of aggressive behavior.^16^ Updated expert recommendations from the MLA Consortium emphasize the importance of integrated morphologic, immunophenotypic, and molecular features in establishing a diagnosis and note current areas of diagnostic uncertainty.^17^

MLA can be recognized as such based on a unique constellation of histomorphologic features, immunoprofile, and recurrent molecular alterations.^1,2,16,18^ These tumors exhibit mesonephric differentiation and characteristically display diverse architectural growth patterns, frequent eosinophilic colloid-like intraluminal secretions, and absence of squamous and mucinous metaplasia. The patterns include tubular, glandular, ductal, papillary, cribriform, retiform, glomeruloid, and solid. While none of these individual patterns is specific, their mixture is the cardinal diagnostic clue. Cytologically, the tumor cells are mostly cuboidal to low columnar, and ovoid to spindle in solid foci. They have vesicular nuclei that often overlap and occasionally exhibit grooves. An immunohistochemical (IHC) panel aids in the diagnosis. These tumors show positivity for PAX8, GATA3, TTF-1, and luminal CD10.^1,2,7,10,16,19–22^ GATA 3 is reported to be the most sensitive marker, although some tumors show TTF-1 positivity in the absence of GATA3.^11^ An inverse relationship between GATA3 and TTF-1 expression is common.^16^ Importantly, assessment of these markers’ expression should always be reported in the context of morphology as they are not entirely specific for MLAs and have been reported in small proportions of other Müllerian carcinomas.^19,23^ The cells in the majority of tumors are negative for ER and PR, although focal ER can be seen,^1–5^^,8–11,16^ as demonstrated in the present tumor. Intriguingly, the present tumor showed focal thyroglobulin positivity, which represented nonspecific staining and contributed to misdiagnosis. Focal nonspecific thyroglobulin positivity of the luminal colloid-like material contributing to misinterpretation was rarely previously described,^2^ although in the present tumor, focal positivity was observed in the cytoplasm as well as the luminal border. On the molecular level, multiple reports have demonstrated that *KRAS* G12 mutations are the most frequent genetic alterations in MLA, with G12V and G12D being the most common variants.^4–6^^,10,11,18,22,24,25^

MLA is now well recognized among endometrial and ovarian carcinomas. However, its occurrence in the peritoneum remains poorly characterized. To date, only 2 studies have reported extrauterine/extraovarian MLA of peritoneal origin, with a total of 6 reported tumors.^10,11^ Ovarian MLA has shown a strong association with endometriosis. Similarly, reported instances of peritoneal MLA demonstrate this association (4 of 6 tumors reported in the literature, in addition to the present tumor). Thus, extrauterine MLA is now recognized as part of the spectrum of endometriosis-associated carcinomas, alongside clear cell and endometrioid carcinomas.^10,11,20^

Clinically, MLA of the endometrium and ovary is considered an aggressive neoplasm, marked by frequent recurrences and distant metastases, most commonly to the lung.^3,12,16,20^ A comparable behavior has been observed in reported peritoneal MLA (*n* = 7). The mean patient age was 66.7 years (range, 51-82 years). Six out of seven tumors presented at advanced FIGO stages (III or IV) and one tumor at stage II. Pulmonary and hepatic metastases were the most frequent sites (three patients each), followed by lymph nodes, omentum, and pelvic peritoneum (one patient each). Three patients developed recurrences. Three patients died of disease, three were alive with disease, and one had no evidence of disease.^10,11^ These data are summarized in Table 1.

**Table 1.** Clinicopathologic Data of Reported Peritoneal Mesonephric-Like Adenocarcinomas.

		Age	Presence of Endometriosis	Original Diagnosis	FIGO Stage	Metastatic Site	Recurrence	Follow-Up (months)
1	Deolet et al^10^	82	Yes	_	Unknown	N/A	N/A	NED, 8
2	Euscher et al^11^	51	Yes	LG EAC	III	Lymph nodes	Lung	AWD, 220
3		74	No	LG EAC	III	Omentum	Lung, Liver	DOD, 32
4		69	No	LG EAC	IV	Lung/ pleura	Lung	DOD, 45
5		67	Yes	HG EAC	IV	Liver	Unknown	DOD, 6
6		69	Yes	LG EAC	II	N/A	N/A	NED, 5
7	Present tumor	55	Yes	Adenocarcinoma, favor metastatic PTC	IV	Liver, lymph nodes	N/A	AWD

AWD, alive with disease; DOD, dead of disease; NED, no evidence of disease; PTC, papillary thyroid carcinoma; LG EAC, low-grade endometrioid carcinoma; HG EAC, high-grade endometrioid carcinoma.

The unfamiliarity with MLA in the endometrium and ovary, their morphologic overlap with other carcinomas, and the absence of a pathognomonic IHC marker or molecular alteration pose a diagnostic challenge. Consequently, misdiagnoses are frequent, with the main diagnostic dilemma lying in distinguishing MLA from mesonephric carcinoma and Müllerian carcinomas, particularly endometrioid adenocarcinoma .^2–5^^,7,8,11,12,16,19,22^ The peritoneal location of the present tumor added further ambiguity, raising the possibility of nongynecologic carcinomas. In the study by Euscher et al, all tumors of peritoneal MLA were misdiagnosed as endometrioid adenocarcinoma (4 as low-grade and 1 as high-grade endometrioid adenocarcinoma).^11^

Among the main differential diagnoses for MLA is mesonephric carcinoma. The morphology, ER/PR negativity, positivity for GATA3 and TTF-1, and *KRAS* mutation all mimic mesonephric carcinoma. The key to avoiding misdiagnosis of MLA as mesonephric carcinoma is appreciation of an unusual location for mesonephric carcinoma, with absence of a potential connection to the trajectory of the mesonephric duct's embryologic course (lateral walls of cervix or vagina, broad ligament, mesosalpinx, or ovarian hilus), the presence of Müllerian lesions (eg, endometriosis),^2,3^ and the co-occurrence of Müllerian-type mutations (*PIK3CA*, *PTEN*) alongside *KRAS* mutations.^26^ Diffuse TTF-1 staining favors MLA.^19^ On the other hand, the presence of benign mesonephric remnants would also aid in the diagnosis of mesonephric carcinoma.^16^

Other Müllerian carcinomas arising in extrauterine locations may enter the differential diagnosis. Endometrioid adenocarcinoma is the most encountered diagnostic challenge. The combination of the variety of growth patterns together with nuclear features is not typical of endometrioid adenocarcinoma. The presence of squamous or mucinous differentiation or MMR deficiency favors endometrioid adenocarcinoma over MLA.^2^ In its classic form, MLA is negative for ER/PR and positive for TTF-1 and GATA3, often with an inverse expression pattern. CD10 positivity is least specific and should be interpreted with caution. PR negativity is more valuable than ER, as it is almost always negative, whereas focal ER is occasionally observed.^2,3,16^

Clear cell carcinoma can display admixture of architectural patterns—most commonly tubulocystic, papillary, and solid—frequent association with endometriosis and double ER/PR negativity. Helpful features favoring clear cell carcinoma include the limited diversity of the architecture, high-grade nuclear features, lack of spindle cell morphology, presence of stromal hyalinization within papillae, hobnailed cells, clear cytoplasm, and high-grade nuclei. Additionally, positivity for Napsin A and HNF1B, together with negativity for GATA3 and TTF-1, supports a diagnosis of clear cell carcinoma over MLA.^2,4,27^

High-grade serous carcinoma can be distinguished from MLA by greater nuclear atypia, lack of the same extent of architectural heterogeneity, mutation-type p53, and typically block-type staining of p16.^2,3.4^ On a cautionary note, aberrant p53 expression in MLAs has been rarely reported, most often as a co-occurrence with high-grade serous carcinoma, and a single reported tumor described as harboring *TP53* as the sole driver mutation.^11^

Carcinosarcoma can enter a differential diagnosis due to solid areas with spindle nuclei observed in many MLAs. However, severe nuclear atypia with aberrant p53 expression characteristic of carcinosarcomas are not typical features of MLA.^2,4^ A rare tumor has been reported representing mesonephric-like carcinosarcoma with chondrosarcomatous elements.^28^ Given that areas of spindle cells are common in MLA, it is prudent that the diagnosis of mesonephric-like carcinosarcoma be reserved for tumors with heterologous elements.^2^

A contributing factor to misdiagnosis of MLA as other Müllerian carcinomas is the fact that in some instances, MLA may be admixed with other histotypes, including borderline tumors (serous, endometrioid, seromucinous), endometrioid adenocarcinoma, low-grade serous carcinoma, clear cell carcinoma, mixed carcinomas, and carcinosarcoma.^11^

Papillary thyroid carcinoma, either metastatic or malignant transformation of struma ovarii, can mimic MLAs due to papillary architecture, papillary thyroid carcinoma-like nuclear features, and PAX8/TTF-1 positivity. However, papillary thyroid carcinoma displays predominant papillary architecture without heterogeneity of growth patterns, a full spectrum of characteristic nuclear features, frequent psammoma bodies, positive thyroglobulin, and typically positive BRAF IHC and/or *BRAF* mutation. A potential diagnostic pitfall is focal nonspecific thyroglobulin expression in MLA, as observed in the present tumor and rarely reported in the literature,^2^ which can lead to erroneous interpretation. *RAS* mutations occur mainly in the follicular-patterned thyroid lesions, including follicular variant of papillary thyroid carcinoma. Of these, *KRAS* is least common and, in a classic subtype of papillary thyroid carcinoma, is exceptionally rare.^29,30^ In the present tumor, the presence of *KRAS* mutation combined with peritoneal metastasis and normal adnexa and thyroid without cervical lymph node involvement, effectively ruled out papillary thyroid carcinoma.

Another interesting feature of the present tumor was the result of the molecular classifier assay (CancerTYPE ID), which is designed to aid in the identification of carcinomas of unknown primary by comparing tumor gene expression profiles with a comprehensive reference database. In this instance, the assay assigned a high probability of renal cell carcinoma. Although the precise basis for this result cannot be determined, it is biologically plausible that the classifier captured shared aspects of gene-expression programs associated with mesonephric differentiation, which have developmental links to the nephric system. MLA exhibits mesonephric differentiation that may overlap with transcriptional programs present in renal epithelial neoplasms. Given the rarity of MLA and its likely underrepresentation in the classifier's training set, the algorithm may have assigned renal cell carcinoma as the closest high-confidence match. Accordingly, results from expression-based molecular classifiers should be interpreted with caution and in conjunction with morphologic, immunophenotypic, molecular, and clinical–radiologic findings.

## Conclusion

In conclusion, MLA shows a strong association with endometriosis, rendering the peritoneum a potential site of tumor origin. Limited awareness of this entity, combined with its occurrence in the peritoneum, poses a significant diagnostic challenge. Pathologists should be aware of this entity as a rare type of endometriosis-associated malignancy.
